# Serum Albumin and Glycemic Variability Could Contribute to Diabetic Retinopathy Progression by Regulating Chronic Inflammatory Pathways

**DOI:** 10.1155/joph/9673736

**Published:** 2025-12-11

**Authors:** Jack Jonathan Maran, Charisse Y.-J. Kuo, Ilva D. Rupenthal, Rinki Murphy, Odunayo Omolola Mugisho

**Affiliations:** ^1^ Buchanan Ocular Therapeutics Unit, Department of Ophthalmology, New Zealand National Eye Centre, University of Auckland, Auckland, New Zealand, auckland.ac.nz; ^2^ Faculty of Medical and Health Sciences, University of Auckland, Auckland, New Zealand, auckland.ac.nz

**Keywords:** albumin, bariatric surgery, diabetic retinopathy, inflammasome, inflammation

## Abstract

**Purpose:**

Despite managing glycemic levels and other risk factors, individuals with type 2 diabetes (T2DM) may still experience diabetic retinopathy (DR) progression. This study investigated clinical predictors of DR progression in T2DM patients following bariatric surgery.

**Methods:**

This study was a retrospective post hoc analysis of participants from a clinical randomized controlled trial of bariatric surgery patients with T2DM (ACTRN12611000751976) who had complete DR screening and blood data at baseline and 5 years post‐bariatric surgery. Plasma cytokine concentrations were also examined.

**Results:**

DR progression was strongly associated with a postoperative decrease in serum albumin (OR = 0.461, 95% CI: 0.221–0.962, *p* = 0.039). This decrease also predicted postoperative increases in IL‐6 (OR = infinity, sensitivity = 100.00%, *p* = 0.0162) and IL‐1β (OR = 11.250, sensitivity = 81.82%, *p* = 0.0154), known to be linked to NLRP3 inflammasome activation. In addition, individuals who progressed in DR severity showed greater month‐to‐month HbA1c variability compared to stable individuals (mean difference: 0.6583%, 95% CI: 0.1821–1.134%, *p* = 0.0115).

**Conclusions:**

Decreases in serum albumin and increased glycemic variability may influence DR progression through the NLRP3 inflammasome and inflammatory pathways. Further research is needed to clarify the role and mechanism of albumin loss in DR progression.

## 1. Introduction

Diabetic retinopathy (DR) is a visually disabling disease and a leading cause of blindness in the working‐age population [1]. The pathogenesis of DR is thought to be linked to prolonged hyperglycemia and inflammation, causing damage to endothelial cells of capillaries at the blood–retinal interface [2]. These changes result in leaky capillaries, manifesting as retinal hemorrhages, ischemia, and deposition of exudates within the retina [2]. Prolonged ischemia can result in neovascularization within the eye, which may precipitate more hemorrhage and retinal edema, resulting in the death of retinal cells [2]. At the severe stages of the disease, retinal cell death can result in the development of fibrous scar tissues, which can lead to tractional retinal detachments and vision loss [2].

The current mainstay therapy for early DR is pharmacological glycemic control for achieving lower blood glucose levels alongside optimizing vascular risk factors, including dyslipidemia, hypertension, and smoking. During neovascular DR, vascular endothelial growth factor inhibitors (anti‐VEGFs) may be used to inhibit neovascularization, and surgery may be required to remove tractional membranes or vitreous hemorrhage [2]. However, these therapies do not halt DR progression. Several studies have indicated that DR may even progress despite rapid improvement in systemic glucose levels or reduction in body weight [3–6]. In particular, bariatric patients who have DR before surgery pose an interesting subgroup as these patients achieve remission of type 2 diabetes mellitus (T2DM) and improved weight‐related comorbidities such as vascular risk factors. However, counterintuitively, several patients with DR who undergo bariatric surgery often progress in DR severity, suggesting that other factors apart from body weight and serum glucose levels influence DR progression [7].

Recent investigations have indicated that the progression of DR might be because of chronic low‐grade inflammation, which may continue despite glycemic or weight control [2]. However, the molecular associations and pathways that underpin chronic inflammation in DR progression have yet to be fully elucidated. Furthermore, there is a lack of evidence surrounding clinically relevant inflammatory biomarkers that can potentially be used to predict DR progression.

Patients with T2DM are often prioritized for bariatric surgery in order to achieve diabetes remission. Such patients have varying severity of DR prior to surgery, which generally stabilizes or regresses after surgery but may progress in some cases despite improvement, if not remission, of diabetes and other weight loss‐related risk factors [7, 8]. In particular, some investigations have suggested that albumin, bilirubin, alanine transaminase (ALT), glycemic variability, and serum lipids may affect the progression of diabetic microvascular sequelae [9–19]. As such, this study aimed to characterize whether these serum biochemical markers and other clinical indicators could be used to predict DR progression in such patients.

## 2. Methods

### 2.1. Participants and Ethics

This retrospective post hoc analysis was conducted using patients from a bariatric surgery randomized controlled trial (RCT) registered with the Australian New Zealand Clinical Trials Registry, which aimed at evaluating the efficacy of laparoscopic silastic ring Roux‐en‐Y gastric bypass (RYGB) against sleeve gastrectomy for the management of T2DM in obese patients (ACTRN12611000751976). The study followed the tenets of the Declaration of Helsinki, and ethical approval was obtained from the Aotearoa Research Ethics Committee (NTY/11/07/082). The authors of this study obtained clinical data from these patients under a confidentiality agreement, which included DR screening data, blood tests from hospital records, glycated hemoglobin (HbA1c) levels, biometrics, and a medication list at various time points (RM14849). Plasma samples were also available for cytokine analysis (RM11868).

Inclusion criteria were as follows: age between 20 and 55 years, diagnosis of T2DM for at least 6 months, body mass index (BMI) of 35–65 kg/m^2^ for at least 5 years, clinically suitable for an RYGB or sleeve gastrectomy and able to give informed consent. Exclusion criteria were a post‐prandial C‐peptide < 350 pmol/L, pregnancy, type 1 diabetes or secondary diabetes, chronic pancreatitis, oral steroid therapy, smoking, and individuals unsuitable for general anesthesia. Patients without a complete set of DR grading data or blood tests were excluded. Patients were recruited between September 2011 and October 2014.

**Figure 1:**
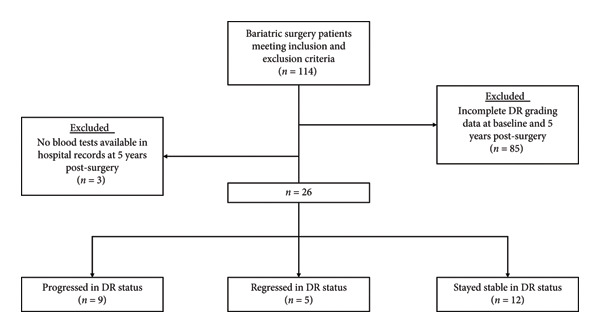
Flowchart of participant inclusion in this study. Patients were ascertained from the ACTRN12611000751976 clinical trial evaluating the efficacy of laparoscopic silastic ring Roux‐en‐Y gastric bypass (RYGB) against sleeve gastrectomy for managing type 2 diabetes mellitus (T2DM) in obese patients. Hundred and fourteen patients meeting the inclusion and exclusion criteria were identified. A further 85 patients were excluded from this study because of not having a complete set of DR grading data at baseline and 5 years post‐surgery. Three additional patients were excluded because of nonavailability of blood tests in their hospital records at 5 years post‐surgery. The remaining 26 patients were included in this study, where 9 progressed, 5 regressed, and 12 stayed stable with regard to DR status.

A total of 114 patients who underwent either an RYGB or a sleeve gastrectomy who fit the inclusion and exclusion criteria were identified. All patients in the original RCT were included for post hoc analysis in this study, as long as the patient’s DR grading and blood test data were available in the clinical records at both baseline and 5 years post‐surgery. The presence of DR or maculopathy at baseline was not a criterion for inclusion or exclusion. Of the original 114 patients, 85 did not have a complete set of DR grading data in both eyes available at both baseline and 5 years post‐surgery. A further three patients had no blood tests available in the hospital medical records at 5 years post‐surgery, leaving a final 26 patients that were included for analysis (Figure 1).

### 2.2. Clinical and Biochemical Data

#### 2.2.1. DR Grading Data

DR photo‐screening was performed presurgery (baseline), 1 year, and 5 years post‐surgery under the New Zealand National Diabetic Retinal Screening Program. Both eyes underwent retinal screening in all patients and were graded according to the New Zealand Diabetic Retinal Screening, Grading, Monitoring and Referral Guidance document between R0 to R5 for retinopathy and M0 to M5 for maculopathy [20]. Retinopathy and maculopathy values were summed for both eyes in each patient. Patients who had increases in retinopathy or maculopathy values from baseline to 1 or 5 years were deemed to have had DR progression within that timeframe. Patients who had decreases in retinopathy or maculopathy values from baseline were deemed to have regressed, while those who had no change were considered stable. Where there was a discrepancy between the direction of change in retinopathy or maculopathy values, maculopathy values took precedence because of macular changes being more visually significant.

Of the 26 patients who had a complete set of DR grading data and blood tests available in hospital records, 9 patients progressed (34.62%), 5 regressed (19.23%) and 12 stayed stable (46.15%) in DR status between baseline and 5 years post‐surgery (Figure 1).

#### 2.2.2. Baseline Anthropometrics, Hospital Blood Tests, and HbA1c Data

Anthropometric data were available at baseline, which included weight, height, BMI, systolic and diastolic blood pressure, and waist circumference. Screening blood test data from hospital records were available for analysis at baseline and 5 years post‐surgery, which included hemoglobin, platelets, white blood cells, albumin, bilirubin, ALT, fasting glucose, HbA1c, triglycerides, cholesterol, high‐density lipoprotein (HDL), cholesterol/HDL ratio, and low‐density lipoprotein (LDL). Additional HbA1c data from clinical records were available at baseline, 3, 6, 9, 12, 18, 24, 36, 48, and 60 months after surgery.

#### 2.2.3. Plasma Cytokine Levels

Serum samples from patients at baseline and 5 years post‐surgery, where available, were assessed for plasma levels of tissue necrosis factor‐alpha (TNF‐α), interleukin‐6 (IL‐6), interleukin‐8 (IL‐8), interleukin‐10 (IL‐10), interleukin‐1‐beta (IL‐1β), interleukin‐18 (IL‐18), and VEGF with a Luminex multiplex assay (Human Premixed Multi‐AnalyteKit, #LXSAHM, R&D Systems, Minneapolis, MN, USA) without diluting plasma samples according to the manufacturer’s instructions. If patients did not have serum samples available at 5 years post‐surgery, serum cytokine levels were measured from plasma samples at 1 year post‐surgery instead.

IL‐6, TNF‐α, and IL‐1β are key cytokines known to be commonly implicated in general inflammation, especially under the influence of nuclear factor kappa‐B (NF‐κB) [21, 22]. Furthermore, VEGF is a driving factor in the progression of DR, with previous studies suggesting that IL‐18 is implicated in increasing VEGF expression during DR [23]. IL‐10 is known to be a key anti‐inflammatory cytokine that has been suggested to ameliorate the progression of DR [24]. As such, the aforementioned cytokines were chosen for analysis in this study.

#### 2.2.4. Statistical Analysis

Biometric data were analyzed with multiple ordinal regression (MOR) and analysis of variance (ANOVA), grouped according to patients who progressed, regressed, or stayed stable in DR status between baseline and 5 years post‐surgery. Hemoglobin, platelets, white blood cells, albumin, bilirubin, ALT, fasting glucose, HbA1c %, triglycerides, cholesterol, HDL, cholesterol/HDL ratio, and LDL levels were converted into a relative percentage change between baseline and 5 years post‐surgery before being similarly analyzed with a generalized linear model (GLM) and MOR to derive odds ratios (OR) and 95% confidence intervals to determine the predictive strength of variables.

Serial HbA1c % values at baseline, 3, 6, 9, 12, 18, 24, 36, 48, and 60 months after surgery were converted to a rolling monthly percentage change compared to the previous time point. Glycemic variability was calculated by the modulus of the average monthly percentage change in HbA1c %. Data were then analyzed with a paired one‐way ANOVA with post‐hoc Tukey’s analysis for multiple comparisons between patients who progressed, regressed, or stayed stable in DR status between baseline and 5 years post‐surgery.

The relative percentage change in albumin and cytokine levels from baseline to after surgery were transformed into categorical variables based on increases (+) or decreases (−) in albumin or cytokine concentration after surgery. A contingency table and Fisher’s exact tests were utilized to measure the association between the annual relative percentage change in albumin and cytokine concentrations. IL‐6 and IL‐1β were additionally analyzed together, where an increase in either of these cytokines was coded as an increase in inflammatory markers, while a decrease in both cytokines was coded as a decrease in serum inflammatory markers.

Data were analyzed using GraphPad 9.3.0 (Prism, USA) and SPSS (IBM, USA). Data are represented as mean ± standard deviation. A *p* value of less than 0.05 was considered significant in all analyses.

## 3. Results

### 3.1. Baseline Anthropometric Data are Not Associated With Changes in DR Status

A total of 26 patients were included in this post hoc analysis. Seventeen patients (62.07%) did not have DR at baseline. Of the other nine (37.93%) patients who had DR at baseline, only two patients had severe disease at baseline, where one patient had Grade 4 maculopathy and retinopathy in both eyes while the other patient had Grade 4 maculopathy in one eye. In addition, one other patient had Grade 2 maculopathy in one eye at baseline. Otherwise, all patients with DR at baseline only had Grade 1 disease without maculopathy. Across the 5‐year follow‐up period, 9 progressed in DR severity, while 5 regressed, and 12 stayed stable. In addition, among the nine patients that progressed, seven of them developed at least Grade 2 disease by 5 years post‐surgery even though four of these patients did not have DR at baseline.

Analysis of baseline data revealed no significant differences in weight, height, BMI, waist circumference, systolic and diastolic blood pressure when analyzed with one‐way ANOVA and MOR. Moreover, average BMI and systolic blood pressure at baseline were higher in the individuals who regressed than those who progressed and stayed stable in DR status. The overall MOR Omnibus test also failed to reach significance (*p* = 0.848), indicating that baseline biometric measurements were not significant predictors of DR progression (Table 1).

**Table 1:** Baseline anthropometric data of bariatric surgery patients.

Anthropometric data	Progressed (*n* = 9)	Regressed (*n* = 5)	Stable (*n* = 12)	*p* value (ANOVA)	*p* value (MOR)
Weight (kg)	119.21 ± 18.47	113.23 ± 13.37	112.55 ± 12.83	0.5539	0.771
Height (m)	1.72 ± 0.13	1.66 ± 0.06	1.69 ± 0.09	0.5345	0.677
BMI (kg/m^2^)	40.65 ± 4.09	41.24 ± 4.32	39.70 ± 4.64	0.7556	0.835
Waist circumference (cm)	131.05 ± 14.38	126.60 ± 12.58	124.91 ± 17.43	0.6597	0.587
Systolic blood pressure (mmHg)	140.00 ± 10.33	143.40 ± 26.12	132.83 ± 17.98	0.4365	0.664
Diastolic blood pressure (mmHg)	84.64 ± 8.61	87.40 ± 6.77	80.25 ± 13.50	0.4120	0.538

### 3.2. Increased Month‐To‐Month Glycemic Variability is Associated With DR Progression

Analysis of serial HbA1c values obtained from patients at baseline, 3, 6, 9, 12, 18, 24, 36, 48, and 60 months after surgery showed that following an initial drop in HbA1c between baseline and 3 months, patients who progressed in DR status generally had greater variability in HbA1c (Supplementary File S1). The cumulative percentage deviation from zero (CD) in glycemic variability between blood collection time points was 57.953, 40.657, and 33.494, respectively, for those who progressed, regressed, and stayed stable in DR status (Supplementary File S1).

Statistical analysis confirmed that, on average, individuals who progressed in DR status were found to have a significantly greater absolute monthly variability in HbA1c compared to those who stayed stable (mean difference: 0.6583%, 95% CI: 0.1821–1.134%, *p* = 0.0115) (Supplementary File S2). HbA1c variability was greater in those who progressed in DR status than in those who regressed as well, but this did not reach statistical significance (*p* = 0.1384) (Supplementary File S2). Individuals who regressed and stayed stable had similar magnitudes of monthly percentage changes in HbA1c (*p* = 0.9678).

### 3.3. A Rise in Serum Albumin Was Associated With a 54% Decrease in the Likelihood of DR Progression

MOR analysis of the percentage change in hemoglobin, platelets, white blood cells, albumin, bilirubin, ALT, fasting glucose, HbA1c %, triglycerides, cholesterol, HDL, cholesterol/HDL ratio, and LDL concentrations between baseline and 5 years post‐surgery revealed that only albumin was a significant predictor of DR progression after adjusting for all 12 other confounding factors (*β* = −0.775, 95% CI: −1.511 to −0.039, *p* = 0.037) (Table 2). The percentage decrease in albumin from baseline to 5 years post‐surgery was inversely related to DR progression (OR = 0.461, 95% CI: 0.221 to 0.962, *p* = 0.039), where the likelihood of DR progression was decreased by approximately 54% per unit rise in serum albumin. In patients who progressed, the mean percentage decrease in albumin concentration was −4.773% (range: −11.9048% to 0.000%), while those who stayed stable or regressed was 3.136% (range: −4.7619%–8.57143%) and 4.924% (range: 0.000%–19.3548%) respectively. Furthermore, the percentage change in albumin was not associated with a change in HbA1c between baseline and 5 years post‐surgery (*r* = −0.0798, 95% CI: −0.453 to 0.317, *p* = 0.6983). All patients had albumin levels within the normal physiological range of 33–52 g/L at baseline and 5 years post‐surgery, except for one individual with an albumin concentration of 31 g/L at baseline.

**Table 2:** MOR and GLM analysis of predictors of DR progression.

Parameter	β	95% CI		*p* value	OR	95% CI		*p* value
		Lower	Upper			Lower	Upper	
Hb	−0.260	−0.697	0.177	0.239	0.771	0.498	1.193	0.243
PLT	0.059	−0.131	0.249	0.541	1.061	0.878	1.282	0.542
WC	0.236	−0.114	0.585	0.181	1.266	0.893	1.795	0.186
Albumin	−0.775	−1.511	−0.039	0.037	0.461	0.221	0.962	0.039
Bilirubin	0.096	−0.06	0.252	0.222	1.101	0.942	1.287	0.227
ALT	−0.052	−0.138	0.034	0.237	0.95	0.871	1.035	0.24
FG	0.088	−0.063	0.238	0.253	1.092	0.939	1.269	0.255
HbA1c	−0.144	−0.422	0.135	0.309	0.866	0.655	1.144	0.311
TG	−0.076	−0.207	0.055	0.256	0.927	0.813	1.057	0.258
C/H ratio	−0.617	−1.489	0.255	0.163	0.54	0.226	1.29	0.165
HDL	−0.355	−0.905	0.195	0.203	0.701	0.405	1.216	0.206
CHOL	0.573	−0.157	1.302	0.121	1.733	0.855	3.678	0.124
LDL	−0.050	−0.124	0.024	0.189	0.951	0.883	1.025	0.189

Abbreviations: ALT, alanine transaminase; C/H ratio, cholesterol to high‐density lipoprotein ratio; CHOL, cholesterol; FG, fasting glucose; Hb, hemoglobin; HbA1c %, glycated hemoglobin percentage; HDL, high‐density lipoprotein; LR, likelihood ratio; LDL, low‐density lipoprotein; OR, odds ratio; PLT, platelets; TG, triglycerides; and WC, white blood cells.

No other parameters on screening blood tests were significant predictors for DR progression. Furthermore, the overall predictive model passed the regression Omnibus test (*p* = 0.002) and accounted for 67.9%–86.1% of the variance in the data (Table 3).

**Table 3:** Statistical parameters for MOR and GLM analysis of predictors of DR progression.

Overall regression Omnibus test	
LR Chi‐Square statistic	32.965
df	13
*p* value	0.002

**Pseudo R-Square**	
Cox and Snell	0.747
Nagelkerke	0.861
McFadden	0.679

**Goodness-of-Fit**	
*p* value (Pearson)	0.052
*p* value (Deviance)	0.996

Abbreviations: df, degrees of freedom; LR, likelihood ratio.

In addition, the mean absolute albumin concentration of all patients who progressed was 39.66 g/L at baseline, which declined by 5.04% to an average of 37.66 g/L at 5 years post‐surgery. The mean absolute albumin concentration of all patients who regressed was 37.20 g/L, which increased by 4.30%–38.80 g/L at 5 years post‐surgery.

### 3.4. Changes in Albumin Concentration Strongly Predict Changes in Serum IL‐6 and IL‐1β Levels in DR Patients

Analysis of the relative percentage change in albumin and individual cytokine concentrations with a Fisher’s exact test revealed that serial changes in IL‐6 (*p* = 0.0162) and IL‐1β (*p* = 0.0154) were significantly predicted by changes in albumin concentration between baseline and after surgery (Table 4). Changes in albumin concentration were not a significant predictor for changes in TNF‐α (*p* = 0.6951), IL‐8 (*p* = 0.411), VEGF (*p* = 0.3783) and IL‐18 (*p* = 0.411) concentrations**.** Furthermore, changes in albumin concentration were significant predictors of both IL‐6 and IL‐1β when both cytokines were analyzed in patients simultaneously (*p* = 0.0188).

**Table 4:** Predictive parameters between albumin and cytokine concentrations.

Predictive parameter	IL‐6	IL‐1β	IL‐6 and IL‐1β
Fisher exact test (*p* value)	0.0162	0.0154	0.0188
OR	Infinity	11.250	10.125
RR	Infinity	4.154	3.808
NNT	2.400	1.902	2.229
LR+	2.571	2.864	2.659
Sensitivity	1.000	0.818	0.818
Specificity	0.611	0.714	0.692
PPV	0.462	0.692	0.692
NPV	1.000	0.833	0.818

Abbreviations: LR+, positive likelihood ratio; NNT, number needed to treat; NPV, negative predictive value; OR, diagnostic odds ratio; PPV, positive predictive value; and RR, relative risk.

Predictive analysis revealed that a decrease in albumin concentration strongly predicted increases in IL‐6 and IL‐1β concentrations, with sensitivities of 100.00% and 81.82%, respectively. Moreover, an increase in albumin concentration was 71.43% specific for predicting decreases in IL‐1β concentration. When IL‐6 and IL‐1β data for patients were analyzed together, a decrease in albumin levels was 81.82% sensitive and 69.23% specific for diagnosing increases in both cytokines together. Increased albumin concentration was associated with a 100.00% and 83.33% chance of a decrease in IL‐6 and IL‐1β, respectively (NPV). In addition, an increase in albumin concentration was associated with an 81.82% chance of a decrease in both IL‐6 and IL‐1β in the same patients. Likewise, a decrease in albumin concentration was associated with a 69.23% chance of an increase in IL‐1β concentration (PPV).

A decrease in albumin concentration was associated with a significant increase in the independent risk of developing increased serum IL‐6 (OR = Infinity, RR = Infinity) and IL‐1β (OR = 11.250, RR = 4.154). Similarly, patients were significantly more likely to have an increase in the level of either or both IL‐6 and IL‐1β (OR = 10.125, RR = 3.808) if their serum albumin levels decreased (Table 4). Further predictive parameters are provided in Table 4, and detailed contingency tables are provided in Supplementary File S3.

## 4. Discussion

In this retrospective cohort study of DR patients, our data indicate that increased HbA1c variability and reduced serum albumin levels were associated with DR progression. In line with several other investigations, our findings suggest that increased visit‐to‐visit HbA1c variability is associated with DR progression as opposed to stagnantly elevated or depleted HbA1c concentrations [15–17]. Typically, elevated glucose levels were thought to be the primary initiator of microvascular damage in DR. However, it is clear that patients may still progress in DR severity despite declines in average glucose levels [3–6].

Consequently, our data showed that changes in serum albumin levels within the normal physiological range were inversely associated with DR progression, even after controlling for 12 other serum biomarkers. This idea has been supported by a few other studies, which similarly show that reduced albumin concentrations may be linked to DR progression [9–11]. In particular, a large‐scale study by Wang et al., which surveyed 45,462 participants, found that serum albumin negatively correlated with DR severity [10]. Supporting this, other investigations have shown that low serum albumin levels may be associated with more advanced diabetic kidney disease [11]. The mechanism underlying low albumin levels in advanced diabetic disease is presently unclear but is thought to be potentially linked to increased urinary losses from hypertensive and vasculopathic comorbidities in patients, resulting in impaired renal function, glomerular damage, and albuminuria [25–27]. Moreover, another study by Fujioka et al. suggested that hypoalbuminemia may result in optic disc edema and increased blood flow velocities within the central retinal artery and vein [28]. This results in increased microvascular damage and leak, which is perceived as a worsening of DR pathology [28, 29]. Another study by Zeng et al. suggested that the relationship between serum albumin and DR was nonlinear [30]. Importantly, a serum albumin level of 38.10 g/L was identified to be a potential inflection point, with levels below this threshold being associated with negative outcomes, while levels above this threshold had no significant association with DR [30]. Similarly, we found that patients who traversed this threshold were found to have a change in the trajectory of their DR status. In patients who progressed, the average albumin concentration plunged below 38 g/L 5 years after surgery. In patients who regressed, average albumin concentration increased to above 38 g/L when measured 5 years after surgery. Taken together, this may suggest that crossing an albumin concentration of 38 g/L may indeed represent a point of inflection for observable clinical impacts of serum albumin on DR [30].

Uniquely, we showed that a reduction in serum albumin levels was a sensitive, strong, and independent risk factor for increased serum IL‐6 (OR = Infinity, RR = Infinity, Sensitivity = 100.00%) and IL‐1β (OR = 11.250, RR = 4.154, Sensitivity = 81.82%) serum levels in DR patients. However, the mechanism of albumin decrease in DR is unclear. One study suggested that albumin may directly leak from blood vessels during angiogenesis, which is known to occur during proliferative DR [31], but presently there is little evidence to suggest that angiogenesis occurs in the body of patients with DR to levels that can significantly affect serum albumin concentrations. Regardless, these findings suggest that preventing albumin decrease may be an effective therapy for avoiding increases in IL‐6 and IL‐1β concentrations in DR patients, with relatively low NNTs of 2.400 and 1.902, respectively. Alongside several other studies that have already established that increased levels of IL‐1β and IL‐6 are associated with DR progression, our findings provide an excellent rationale for the potential involvement of albumin in inflammation during DR progression. This indicates that further investigation into the underlying mechanisms is warranted [32–38].

However, most studies fail to recognize that reduced albumin may also be linked to DR progression because of the intrinsic anti‐inflammatory and antioxidant properties of albumin [11]. Albumin is an essential extracellular antioxidant that regulates glucuronidase and lipid peroxidase activity [39, 40]. Zhang J. et al. highlighted that serum albumin might bind glucuronide conjugates and inhibit oxidative stress by reducing reactive oxygen and nitrogen species (ROS and RNS), where persistent hypoalbuminemia can accelerate oxidative cell damage [11, 41]. Furthermore, alongside protecting cells against oxidant injury, albumin has also been shown to decrease nuclear factor‐kappaB (NF‐κB) activation, which is responsible for cytokine production and the induction of several inflammatory genes [42]. A unique study by Zhang and Frei found that albumin inhibited the expression of vascular cell adhesion molecule‐1 (VCAM‐1) and the adhesion of monocytic THP‐1 cells to human aortic endothelial cells in a dose‐dependent manner [43]. This study also identified that albumin alone strongly inhibited the nuclear translocation and activation of NF‐κB by approximately 90% at physiological concentrations [43]. Accordingly, we found that IL‐6, one of the most NF‐κB‐dependent cytokines, was strongly predicted by changes in serum albumin levels, suggesting that albumin may indirectly modulate the NF‐κB pathway in DR patients [44]. Given that inflammation because of NF‐κB activation is associated with DR progression, albumin may be a potent protective agent against inflammatory and oxidative stress, which are implicated in the pathophysiology of DR [45].

However, an exact molecular mechanism linking albumin, glycemic variability, and DR progression has yet to be elucidated. Within the context of DR, one regulator of chronic inflammation in DR that is of interest in the literature is the NOD‐like receptor protein 3 (NLRP3) inflammasome. The NLRP3 inflammasome is a multimeric protein complex that is responsible for the release of IL‐1β, IL‐18, and pyroptotic cell death in response to pathogen‐associated molecular patterns (PAMPs) or danger‐associated molecular patterns (DAMPs), such as ROS, extracellular potassium, or adenosine triphosphate (ATP) [46]. Interestingly, the NLRP3 inflammasome is thought to become chronically activated in DR and other inflammatory diseases and contributes to the propagation of chronic inflammation [34, 47–51]. Although the mechanism of glycemic variability on DR progression is unclear, a remarkable study by Lee et al. found that acute glucose shifts in both directions increase ROS generation and prominently activate the NLRP3 inflammasome in a ROS‐mitogen‐activated protein kinase (MAPK)‐NF‐κB‐dependent manner [52]. Very few studies have directly assessed the effect of albumin on NLRP3 activation in endothelial and retinal cells. However, one study by Erdei et al. found that albumin prevented NLRP3 activation in endothelial cells exposed to extracellular heme and was associated with a reduction in ROS [53]. Our findings support this theory, where reductions in albumin concentration strongly predicted increases in IL‐1β concentrations, a key product of NLRP3 inflammasome activation. Alongside this, our data indicated that reduced albumin levels were associated with individuals who progressed in DR severity. Taken together, this suggests that maintaining physiological levels of albumin and preventing declines in serum albumin levels may prevent NLRP3 inflammasome activation in DR by reducing ROS, regulating NF‐κB and inflammatory cytokine release, and limiting DR progression. It is, however, important to note that a limiting factor in interpreting our findings in this study was the high rate of exclusion of eligible patients in the original RCT from which the subjects for the present investigation were derived. High rates of exclusion in the original RCT may mean that our present findings may have limited generalizability to larger populations, given the heterogeneity in morbidity in a natural patient population. Further investigations of more heterogeneous patient populations are likely required to improve the translatability of our results.

## 5. Conclusion

Declines in serum albumin concentration are associated with DR progression and increased serum IL‐6 and IL‐1β. Notably, albumin may be a potent inhibitor of ROS, NF‐κB, and the NLRP3 inflammasome in endothelial and retinal cells, which are all pathways thought to be pathologically overactivated in DR. Our findings also provide evidence that elevated glycemic variability may drive DR progression through activating the NLRP3 inflammasome. Therefore, albumin and the NLRP3 inflammasome may be novel therapeutic targets to mitigate DR progression. However, further targeted investigations in animal and human models are required to elucidate the underlying mechanisms for this entirely.

## Consent

The study followed the tenets of the Declaration of Helsinki, and ethical approval was obtained from the Aotearoa Research Ethics Committee (NTY/11/07/082).

## Disclosure

All authors have read and agreed to the published version of the manuscript.

## Conflicts of Interest

The authors declare no conflicts of interest.

## Author Contributions

Idea conception: O.O.M. and J.J.M.; data acquisition: C.Y.K. and O.O.M.; data analysis: J.J.M.; interpretation of data: J.J.M and O.O.M.; manuscript preparation: J.J.M; manuscript revision: J.J.M, C.Y.K, I.D.R, R.M., and O.O.M.; project administration: O.O.M.; funding acquisition: I.D.R, R.M., and O.O.M.

## Funding

O.O.M. was funded by the Neurological Foundation of New Zealand First Postdoctoral Fellowship (Grant number: 2001 FFE), an Auckland Medical Research Foundation (AMRF) Project Grant (Grant number: 1121013), and a Health Research Council Emerging Researcher First Grant (Grant number: 22/546). O.O.M. is currently funded by an AMRF Fellowship (1323001). C.Y.K. was funded by the Vernon Tews Charitable Trust. I.D.R.’s directorship is supported by the Buchanan Charitable Foundation.

## Supporting Information

Additional supporting information can be found online in the Supporting Information section.

## Supporting information


**Supporting Information 1** Figure S1: Figure entailing the serial percentage change in HbA1c % over 5 years from surgery in patients who progressed, regressed, and stayed stable in DR status.

**Supporting Information 2** Figure S2: Figure and ANOVA analysis of the difference in the absolute average monthly percentage change in HbA1c between patients who progressed, regressed, and stayed stable in DR status.

**Supporting Information 3** Figure S3: Contingency tables examining the association between albumin and cytokines before and after surgery.

## Data Availability

The datasets used and analyzed during the current study are available from the corresponding author on reasonable request.
