# Supplementary material for: Serum Albumin and Glycemic Variability Could Contribute to Diabetic Retinopathy Progression by Regulating Chronic Inflammatory Pathways
Source: J Ophthalmol. 2025 Dec 11;2025:9673736. doi: 10.1155/joph/9673736 (PMC12767037; doi:10.1155/joph/9673736)
Supplement: Supplementary file 1 — Supporting Information 1 Figure S1: Figure entailing the serial percentage change in HbA1c % over 5 years from surgery in patients who progressed, regressed, and stayed stable in DR status. [file JOPH-2025-9673736-s001.docx]

# Supplementary File 1

Supplementary File 1: Serial percentage change in HbA1c % between measurement time points. HbA1c % levels were measured at baseline, 3, 6, 9, 12, 18, 24, 36, 48 and 60 months after surgery. The serial percentage change in HbA1c % was calculated for each time point compared to the previous blood collection time point. The cumulative deviation (CD) is represented by the sum of absolute percentage changes in HbA1c % across all blood collection time points. Individuals who progressed had a higher CD than those who regressed or stayed stable in DR status.
