# Supplementary material for: Serum Albumin and Glycemic Variability Could Contribute to Diabetic Retinopathy Progression by Regulating Chronic Inflammatory Pathways
Source: J Ophthalmol. 2025 Dec 11;2025:9673736. doi: 10.1155/joph/9673736 (PMC12767037; doi:10.1155/joph/9673736)
Supplement: Supplementary file 2 — Supporting Information 2 Figure S2: Figure and ANOVA analysis of the difference in the absolute average monthly percentage change in HbA1c between patients who progressed, regressed, and stayed stable in DR status. [file JOPH-2025-9673736-s002.docx]

# Supplementary File 2

Supplementary File 2: Absolute average monthly percentage change in HbA1c. HbA1c values were available at baseline, 3, 6, 9, 12, 18, 24, 36, 48 and 60 months after surgery. The average monthly change in HbA1c was calculated for each time point compared to the previous measurement time point, divided by the number of months between the time points. Data between baseline and 3 months were excluded from analysis as all groups experienced a similar large decline in HbA1c, which confounded statistical analysis. This was supported by analysis with ANOVA, which showed no significant difference between the magnitude of HbA1c decline between baseline and 3 months among patients who progressed, regressed, and stayed stable in DR status (p = 0.6719). Individuals who progressed in DR had a statistically significantly greater absolute monthly relative change in HbA1c than those who stayed stable (p = 0.0115). Individuals who progressed in DR status also had a greater absolute monthly relative change in HbA1c than those who regressed, but this did not reach statistical significance (p = 0.1384). Those who regressed and stayed stable had similar average monthly percentage changes in HbA1c, with no significant differences (p = 0.9678). Data were analyzed by a paired one-way ANOVA test with a Geisser-Greenhouse correction for unequal variability of differences and Tukey’s post hoc test for multiple comparisons.
