# Supplementary material for: Serum Albumin and Glycemic Variability Could Contribute to Diabetic Retinopathy Progression by Regulating Chronic Inflammatory Pathways
Source: J Ophthalmol. 2025 Dec 11;2025:9673736. doi: 10.1155/joph/9673736 (PMC12767037; doi:10.1155/joph/9673736)
Supplement: Supplementary file 3 — Supporting Information 3 Figure S3: Contingency tables examining the association between albumin and cytokines before and after surgery. [file JOPH-2025-9673736-s003.docx]

# Supplementary File 3

**Supplementary File 3:** **Contingency tables for the association between albumin and cytokines before and after surgery**

| **IL-6 (p = 0.0162)** | | Cytokine increase (+) | Cytokine decrease (-) |
| --- | --- | --- | --- |
| Albumin | Decrease (+) | 6 | 7 |
|  | Increase (-) | 0 | 11 |
| **IL-1β (p = 0.0154)** | | Cytokine increase (+) | Cytokine decrease (-) |
| Albumin | Decrease (+) | 9 | 4 |
|  | Increase (-) | 2 | 10 |
| **IL-18** (p = 0.4110) | | Cytokine increase (+) | Cytokine decrease (-) |
| Albumin | Decrease (+) | 6 | 7 |
|  | Increase (-) | 3 | 9 |
| **VEGF** (p = 0.3783) | | Cytokine increase (+) | Cytokine decrease (-) |
| Albumin | Decrease (+) | 5 | 8 |
|  | Increase (-) | 2 | 10 |
| **IL-8** (p = 0.4110) | | Cytokine increase (+) | Cytokine decrease (-) |
| Albumin | Decrease (+) | 6 | 7 |
|  | Increase (-) | 3 | 9 |
| **TNF-α** (p = 0.6951) | | Cytokine increase (+) | Cytokine decrease (-) |
| Albumin | Decrease (+) | 7 | 6 |
|  | Increase (-) | 5 | 7 |
| **IL-6 and IL-1β (p = 0.0188)** | | Increase in at least one of the cytokines (+) | Decrease in both cytokines (-) |
| Albumin | Decrease (+) | 9 | 4 |
|  | Increase (-) | 2 | 9 |

25 of 26 patients had cytokine data at baseline, 1 year and 5 years post-surgery that was available for analysis. If data were unavailable at 5 years post-surgery, plasma samples from 1-year post-surgery were analyzed instead. Extreme outliers were excluded from analysis through outlier analysis of annualized values.
